# Sequencing of the complete mitochondrial genomes of eight freshwater snail species exposes pervasive paraphyly within the Viviparidae family (Caenogastropoda)

**DOI:** 10.1371/journal.pone.0181699

**Published:** 2017-07-25

**Authors:** Ju-Guang Wang, Dong Zhang, Ivan Jakovlić, Wei-Min Wang

**Affiliations:** 1 Key Lab of Freshwater Animal Breeding of the Ministry of Agriculture, College of Fisheries, Huazhong Agricultural University, Wuhan, PR China; 2 Key Lab of Agricultural Animal Genetics, Breeding and Reproduction of the Ministry of Education, Freshwater Aquaculture Collaborative Innovation Center of Hubei Province, Wuhan, PR China; 3 Institute of Hydrobiology, Chinese Academy of Sciences, Wuhan, PR China; 4 University of Chinese Academy of Sciences, Beijing, PR China; 5 Bio-Transduction Lab, Wuhan Institute of Biotechnology, Wuhan, PR China; 6 Collaborative Innovation Center for Efficient and Health Production of Fisheries in Hunan Province, Changde, China; Saint Mary's University, CANADA

## Abstract

Phylogenetic relationships among snails (Caenogastropoda) are still unresolved, and many taxonomic categories remain non-monophyletic. Paraphyly has been reported within a large family of freshwater snails, Viviparidae, where the taxonomic status of several species remains questionable. As many endemic Chinese viviparid species have become endangered during the last few decades, this presents a major obstacle for conservation efforts. Mitochondrial genomes (mitogenomes) carry a large amount of data, so they can often provide a much higher resolution for phylogenetic analyses in comparison to the traditionally used molecular markers. To help resolve their phylogenetic relationships, the complete mitogenomes of eight Chinese viviparid snails, *Viviparus chui*, *Cipangopaludina chinensis*, *C*. *ussuriensis*, *C*. *dianchiensis* (endangered), *Margarya melanioides* (endangered), *M*. *monodi* (critically endangered), *Bellamya quadrata* and *B*. *aeruginosa*, were sequenced and compared to almost all of the available caenogastropod mitogenomes. Viviparidae possess the largest mitogenomes (16 392 to 18 544 bp), exhibit the highest A+T bias (72.5% on average), and some exhibit unique gene orders (a rearrangement of the standard MYCWQGE box), among the Caenogastropoda. Apart from the Vermetidae family and Cerithioidea superfamily, which possessed unique gene orders, the remaining studied caenogastropod mitogenomes exhibited highly conserved gene order, with minimal variations. Maximum likelihood and Bayesian inference analyses, used to reconstruct the phylogenetic relationships among 49 almost complete (all 37 genes) caenogastropod mitogenomes, produced almost identical tree topologies. Viviparidae were divided into three clades: a) *Margarya* and *Cipangopaludina* (except *C*. *ussuriensis*), b) *Bellamya* and *C*. *ussuriensis*, c) *Viviparus chui*. Our results present evidence that some *Cipangopaludina* species (*dianchiensis* and *cathayensis*) should be renamed into the senior genus *Margarya*. The phylogenetic resolution obtained in this study is insufficient to fully resolve the relationships within the ‘b’ clade, but if *C*. *chinensis* proves to be a valid representative of the genus, *C*. *ussuriensis* may have to be reassigned a different genus (possibly *Bellamya*, or even a new genus). Non-monophyly also remains pervasive among the higher (above the family-level) Caenogastropod taxonomic classes. Gene order distance matrix produced a different phylogenetic signal from the nucleotide sequences, which indicates a limited usability of this approach for inferring caenogastropod phylogenies. As phenotypic homoplasy appears to be widespread among some viviparid genera, in order to effectively protect the rapidly diminishing endemic Viviparid populations in China, further detailed molecular phylogenetic studies are urgently needed to resolve the taxonomic status of several species.

## Introduction

The majority of all living gastropods are classified within the Caenogastropoda (Mollusca: Gastropoda) clade, divided into approximately 136 extant families, mostly comprised of sea and freshwater snails [1]. Viviparidae (Gray 1847) are a caenogastropod family of almost globally (except Antarctica and South America) distributed operculate freshwater snails that includes approximately 150 species divided into 31 genera [2, 3]. Traditionally, based on morphological characteristics, Chinese Viviparidae were taxonomically classified into over 70 species, divided into nine genera: *Viviparus*, *Filopaludina*, *Angulyagra*, *Mekongia*, *Rivularia*, *Siamopaludina*, *Margarya*, *Bellamya* and *Cipangopaludina* [4, 5]. The last two are the most speciose Chinese viviparid genera. Among these, the entire *Margarya* genus (Nevill 1877) [6–8] and *Cipangopaludina dianchiensis*, described as a new species in 1990 [9], are endemic to Yunnan plateau (S-W China) [8]. Rapid industrial development and urbanisation during the last 25 years have been associated with equally rapid environmental degradation in China, leading to perturbations in the viviparid abundance and species composition in habitats throughout the entire country [10]. As a result, several endemic Chinese viviparid species have become (critically) endangered [11, 12]. In order to protect and restore the populations of these freshwater snails, their taxonomy, phylogeny, life history and genetics need to be much better understood. Over the past 20 years, several studies of gastropod phylogenies demonstrated that molecular biology and traditional morphological approaches produce contradictory results, as paraphyly was observed for several viviparid genera when molecular data were applied [8, 13–16]. Furthermore, the phylogeny of the entire Caenogastropoda clade remains poorly resolved [1, 17]. The availability of genetic data for molecular phylogenetic studies is crucial for biodiversity conservation [18], and mitochondrial phylogenomics is often capable of providing a phylogenetic resolution superior to the traditionally used single molecular markers [19–21]. Although this approach is not without limitations [22], mitochondrial phylogenomics has successfully addressed a broad range of phylogenetic questions [20], including those among the Gastropoda [17, 21, 23–25]. As mitogenomes of some molluscan groups are known to exhibit extensive genome length and architecture instability [26], besides its use as a phylogenetic marker, the availability of complete mitogenomic sequences for different organisms provides a good model system for decoding the mechanisms of mitogenomic evolution [24, 27]. Only one complete viviparid mitogenome has been published so far, *Cipangopaludina cathayensis* [28], and two more are currently (Feb 2017) available from the GenBank: *Bellamya quadrata* and another *C*. *cathayensis* (both unpublished). This scarcity of publicly available mitogenomic data hampers the understanding of viviparid phylogenetics, which presents a major obstacle for the conservation of rapidly shrinking endemic Chinese viviparid populations. Hence, we have sampled viviparid populations at three locations covering nearly the entire length of the SW-NE diagonal across China. Following taxonomic determination, we have sequenced, annotated and characterised the complete mitogenomes of all eight sampled Viviparidae species. Finally, we have used these, as well as almost all of the publicly available, mitogenomic sequences to reconstruct the phylogenetic relationships within the Viviparidae and the entire Caenogastropoda class. Thus the objective of this study was to generate a large amount of mitogenomic data and use them to attempt to understand better the evolution of Caenogastropod mitochondrial genomes, as well as the taxonomic and phylogenetic relationships within the Viviparidae family.

## Materials and methods

### Ethics statement

Due to dwindling numbers, the sale of *Margarya* species is forbidden in China, but sampling for scientific purposes currently does not require a special permit, whereas the remaining species are currently not protected by law in China. Sampling and research in Dianchi Lake were permitted by the Dianchi Lake Administration Department of the local Kunming (Yunnan province) government. As the remaining two sampling locations, in Anyang and Fuyuan (see the following section for details), were carried out in public rivers and didn't involve any endangered species, and as the Chinese law currently does not require specific permissions for sampling of molluscs in such circumstances, permits weren’t required for these two locations. The permit number of the Huazhong Agricultural University for conducting animal experiments is SCXK(Hubei)2015-0019.

### Samples and DNA extraction

We have selected three sampling spots along the entire SW-NE diagonal across China (Table 1, S1 Fig), associated with three different climate zones: 1. Lake Dianchi in Kunming city, Yunnan province, SW corner of China (south subtropical climate zone); 2. Zhangwei River, near Anyang city, Henan province, central China (warm temperate climate zone); 3. Amur River, about 1 km northwest of Fuyuan city, Heilongjiang province, NE corner of China (middle temperate climate zone). At every location we have collected benthic organisms along 50 meters of shore using hand-held nets. After selecting all of the viviparid snails found in the sample, the remaining organisms were returned to the water. At the Dianchi location, because some of the species have become very rare [12], we have also relied on local fishermen to obtain as many species as possible. However, the sampling was not guaranteed to provide a comprehensive representation of viviparid species at any of the field sites. Snails were transported alive to our lab in Wuhan, where they were taxonomically determined based on their morphological features (S1 Table) as described before [4, 9, 29, 30]. Five different species were found in Lake Dianchi (*Margarya melanioides*, *M*. *monodi*, *Cipangopaludina dianchiensis*, *C*. *chinensis* and *Bellamya aeruginosa*), three in the Zhangwei River (*C*. *chinensis*, *B*. *aeruginosa* and *B*. *quadrata*), and two in the Amur River (*C*. *ussuriensis* and *Viviparus chui*), adding up to eight different species. One adult snail specimen belonging to each species was selected for sequencing (Table 1).

**Table 1:** Sampling and specimen details.

Species	Location	Date	Geographic coordinates	No.	Specimen voucher	GenBank	Size (bp)	A+T %
***Margarya melanioides***	Lake Dianchi	Sep 2015	N 24°50’4.73”, E 102°40’24.55”	1	KM-20150920-Mme-1	KY196442	17 083	71.7
***Margarya monodi***				1	KM-20150918-Mmo-1	KY196441	17 051	71.3
***Cipangopaludina dianchiensis***				2	KM-20150920-Cdi-1	KY200976	17 044	71.6
***Cipangopaludina chinensis***	Zhangwei River	Jun 2016	N 36°03’99.09”, E 114°63’47.43”	a	AY-20160612-Cch-2	KY679831	17 009	71.4
***Bellamya aeruginosa***				a	AY-20160612-Ba-1	KY679832	17 013	74.9
***Bellamya quadrata***				a	AY-20160612-Bq-5	KY679834	18 544	74.1
***Cipangopaludina ussuriensis***	Amur River	Aug 2016	N 48°22'13.47'', E 134°16'2.03''	a	FY-20160813-Cu-1	KY679830	16 596	74.6
***Viviparus chui***				a	FY-20160813-Vc-1	KY679829	16 392	69.4

Date = sampling date; No. = the number of specimens obtained, where “a” indicates that species was abundant; GenBank = GenBank accession number; Size = genome size in base pairs; A+T % = the A+T contents of each genome in %.

### PCR amplification and sequencing

The total DNA was extracted from the foot muscle using Mollusc DNA Kit (Omega, USA) according to the manufacturer's recommended protocol. Publicly available sequences were used to determine the parts of *COX1*, *12S*, *16S*, *Cytb* and *COX3* genes conserved across different caenogastropod species, which were then used to design five primer pairs (S2 Table) for amplification and sequencing of fragments of these genes in all eight species. Based on these newly sequenced fragments, species-specific primers were designed to amplify the complete mitogenome by long-range PCR. The 50 μL volume constituted of 1 U of KOD FX polymerase (Toyobo, Japan), 2.5 μL (approximately 100 ng) of DNA, 25 μL 2x PCR buffer for KOD FX, 10 μL of 2mM dNTPs, and 1.5 μL of each 10 pmol primer. PCR amplification was performed under the following procedure: denaturation at 94°C for 2 min, followed by 35 cycles of 10 s at 98°C, with the annealing temperature (Tm) adjusted to suit the specific primer (generally at Tm—5°C), extension step (68°C) time set to 1 min per Kb of the expected product size (1 to 5 min in total), and the final 5 min extension at 68°C. The PCR products were resolved by electrophoresis on 1.0% agarose gel and sequenced by Tsingke company (Wuhan, China) using primer walking strategy [31].

### Sequence analysis

The sequencing results were subjected to quality control: chromatograms were visually inspected and low-quality reads accordingly manually deleted at both ends of sequences. Sequences were then BLASTed to confirm that the amplicon is the actual target. Mitogenomes were assembled manually in a stepwise manner with the help of DNAstar 5.0 software [32] and annotated in Geneious program [33] using two available viviparid mitogenome sequences as references: *Bellamya quadrata* (NC_031850, unpublished) and *Cipangopaludina cathayensis* (KM503121) [28]. Protein-coding genes (PCGs) were determined by finding the ORFs employing the invertebrate mitochondrial genetic code (transl_table = 5) [34] and checking the nucleotide alignments against the reference genomes in Geneious. Transfer RNA genes were identified using the results of both ARWEN [35] and MITOS [36] analyses. Similarly, rRNAs (12S and 16S) were identified using MITOS and alignments to closely related references in Geneious. Base composition was computed using MEGA 5 [37]. Tables with statistics for mitogenomes were generated using a newly developed GUI-based program, MitoTool (https://github.com/dongzhang0725/MitoTool). Rearrangement events in the mitogenomes and pairwise comparisons of gene orders were analysed by CREx web tool [38] utilizing breakpoint dissimilarity measurement. The annotated mitogenome sequences were deposited in GenBank (see Table 1 for accession numbers). The methodology is also available from http://dx.doi.org/10.17504/protocols.io.h43b8yn.

### Phylogenetic analyses

In order to infer phylogenetic relationships among the studied species, we have retrieved all three complete viviparid (Gastropoda: Caenogastropoda: Viviparidae) mitogenomes available in the GenBank. As many new mitogenomes have been sequenced and deposited into the GenBank since the last comprehensive mitochondrial phylogenomic study [17] of the largest and most diverse gastropod group, Caenogastropoda clade, we have included nearly all of the publicly available caenogastropod mitogenomes in the analysis. Exceptions are Conidae and Muricidae families, both represented by several species in the GenBank, which were found to be monophyletic by previous studies applying mitochondrial phylogenomics approach [23, 39]; and thus only one sequence was selected to represent each family. *Glyphostoma* sp. (KX263260, Clathurellidae family) sequence was missing the *NAD5* gene, and *Ilyanassa obsoleta* (DQ238598, Nassariidae) sequence possessed a non-standard, very likely misannotated, *NAD2* gene; therefore we removed these two sequences from the dataset. All of the remaining species represented in the GenBank were included in the dataset; comprising 39 sequences, 38 species, 26 families and 15 superfamilies (Table 2). Two sequences from groups basal to Viviparidae, Vetigastropoda and Neritimorpha, were used as outgroups. Along with the eight newly sequenced Viviparidae, a total of 49 sequences were used in the phylogenetic analysis.

**Table 2:** Mitochondrial genome sequences retrieved from GenBank for this study.

Superfamily	Family	Species	Accession ID	Reference
Ampullarioidea	Viviparidae	*Cipangopaludina cathayensis*	KM503121	[28]
		*Cipangopaludina cathayensis*	KX688549	Unpublished
		*Bellamya quadrata*	NC_031850	Unpublished
	Ampullariidae	*Pomacea canaliculata*	NC_024586	[47]
		*Pomacea maculata*	NC_027503	[48]
		*Marisa cornuarietis*	NC_025334	[49]
Muricoidea	Muricidae	*Menathais tuberosa*	KU747972	[50]
	Babyloniidae	*Babylonia lutosa*	NC_028628	[51]
		*Babylonia areolata*	HQ416443	[51]
Olivoidea	Olividae	*Amalda northlandica*	NC_014403	[52]
	Volutidae	*Cymbium olla*	EU827199	[25]
Buccinoidea	Buccinidae	*Neptunea arthritica*	KU246047	[53]
		*Buccinum pemphigus*	NC_029373	Unpublished
		*Volutharpa perryi*	NC_028183	Unpublished
	Nassariidae	*Nassarius reticulatus*	EU827201	[25]
		*Varicinassa variciferus*	KM603509	Unpublished
Vermetoidea	Vermetidae	*Dendropoma gregarium*	NC_014580	[26]
		*Ceraesignum maximum*	NC_014583	[26]
		*Eualetes tulipa*	NC_014585	[26]
		*Thylacodes squamigerus*	NC_014588	[26]
Truncatelloidea	Pomatiopsidae	*Oncomelania hupensis*	FJ997214	Unpublished
	Hydrobiidae	*Potamopyrgus estuarinus*	GQ996415	[54]
		*Potamopyrgus antipodarum*	GQ996416	[54]
Conoidea	Clavatulidae	*Fusiturris similis*	EU827197	[25]
	Turridae	*Lophiotoma cerithiformis*	DQ284754	[55]
	Terebridae	*Oxymeris dimidiata*	EU827196	[25]
	Conidae	*Africonus borgesi*	NC_013243	[25]
Littorinoidea	Littorinidae	*Littorina saxatilis*	KU952094	[56]
Tonnoidea	Ranellidae	*Cymatium parthenopeum*	EU827200	[25]
	Cassidae	*Galeodea echinophora*	KP716635	[17]
Naticoidea	Naticidae	*Naticarius hebraeus*	KP716634	[17]
Cancellarioidea	Cancellariidae	*Cancellaria cancellata*	NC_013241	[25]
Cerithioidea	Pachychilidae	*Tylomelania sarasinorum*	NC_030263	[57]
	Turritellidae	*Turritella bacillum*	NC_029717	Unpublished
	Semisulcospiridae	*Semisulcospira libertina*	NC_023364	[58]
Abyssochrysoidea	Provannidae	*Provanna sp*.	KM675481	Unpublished
		*Ifremeria nautilei*	KC757644	[59]
Cyclophoroidea	Cochlostomatidae	*Obscurella hidalgoi*	KP716638	[17]
Stromboidea	Strombidae	*Strombus gigas*	KM245630	[60]
Vetigastropoda		*Haliotis rubra*	AY588938	[61]
Neritimorpha		*Pleuropoma jana*	KU342666	[39]

Higher taxonomic levels (superfamily and family) are indicated in the two columns on the left.

A Fasta file with the nucleotide sequences for all 37 genes (13 PCGs, 2 rRNAs and 22 tRNAs) was extracted from the GenBank files using MitoTool. All genes were aligned in batches using MAFFT 7.149 [40] in conjunction with another newly developed GUI-based software, BioSuite (https://github.com/dongzhang0725/BioSuite), wherein codon-alignment mode was used for the PCGs, and normal alignment mode for the rRNAs and tRNAs. BioSuite was then used to concatenate these alignments and generate input files for the phylogenetic analyses conducted using RaxML GUI (maximum likelihood—ML) [41, 42] and MrBayes 3.2.1 (Bayesian inference—BI) [43]. Saturation analysis results, conducted using DAMBE program [44], indicate low saturation for all three codon positions. Based on the Akaike Information Criterion implemented in ModelGenerator v0.8527 [42, 45], GTR+I+G was chosen as the optimal model of nucleotide evolution. Maximum likelihood analysis was performed using an ML+rapid bootstrap algorithm and BI analysis was performed using the default settings with 1x10^7^ MCMC generations (average SD of split frequencies = 0.000955). Statistical support for the nodes was expressed as bootstrap support (1000 replicates) for the ML and posterior probability (pp) for the BI analysis. Finally, the resultant trees and gene orders were annotated and visualised by iTOL [46] using several dataset files generated by MitoTool.

## Results and discussion

### Sampled species

Among the eight sampled species, one is critically endangered (*Margarya monodi*) whilst two are endangered (*M*. *melanioides* and *Cipangopaludina dianchiensis*) [11]. As expected, since the majority of the endangered Chinese Viviparidae (mostly *Margarya* species) are endemic to the Yunnan province [5], all three endangered species were found only in Lake Dianchi, the largest (298 km^2^, 1866 m elevation) freshwater lake in Yunnan province [62]. In the 1980s, the lake was inhabited by eleven viviparid freshwater snail species, divided into four genera: *Cipangopaludina*, *Margarya*, *Angulyagra* and *Bellamya* [63]. Historical data (dating to 1949) indicate that snails belonging to *Margarya* genus were harvested from the Dianchi lake in large quantities as food for human consumption [10]. However, as the lake is located on the outskirts of the largest city in Yunnan province, Kunming, a tremendous increase in the wastewater discharge into the lake, resulting from rapid industrial development and fast urban population growth during the last 25 years, as well as the lack of environmental awareness and enforcement of environmental protection laws, has led to a severe water pollution and lake eutrophication [62, 64, 65]. This has resulted in the loss of about 75% of mollusc populations and in the majority of Viviparidae species becoming locally endangered or extinct [10]. Pollution in the Zhangwei River is much less severe [66] than in Dianchi Lake, whereas in the Amur River it is rather mild [67], thus freshwater snail populations in these two rivers are currently stable. However, some viviparid species appear to be much more sensitive to pollution than others: some *Bellamya* and *C*. *chinensis* species are still abundant both in Dianchi Lake and elsewhere in rather severely polluted waters in China, whereas *Margarya* species and *C*. *dianchiensis* are very sensitive to pollution and their numbers are rapidly dwindling [12]. This was reflected in the results of our sampling (Dianchi), where we could find only one specimen of each *Margarya* species and two *C*. *dianchiensis* specimens (Table 1). These large differences in pollution tolerance are rather intriguing, given the phylogenetic proximity of these species, and therefore warrant further studies.

### General features of the mitogenomes

**Fig 1:**
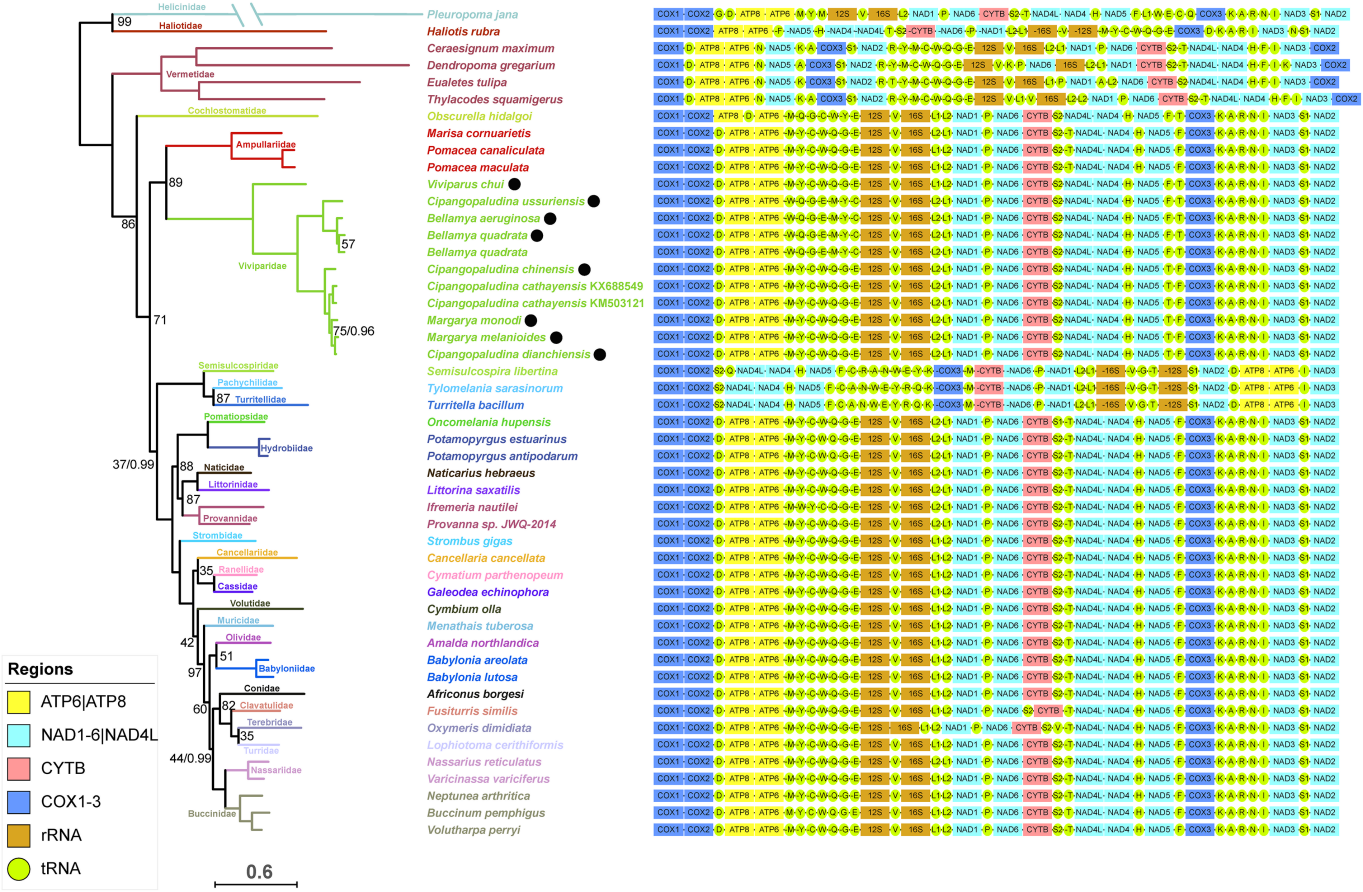
Phylogenetic relationships and gene order. Phylogenetic relationships among the majority of the available caenogastropod mitogenomic sequences (Table 2) based on nucleotide sequences of all 37 genes. *Haliotis rubra* and *Pleuropoma jana* were used as outgroups. Numbers on the nodes correspond to ML bootstrap support (left, expressed as 0–100) and BI posterior probabilities (right, expressed as 0–1.0). Only values below 100/1.0 are depicted. Gene order is shown next to each sequence.

The lengths of the eight newly sequenced mitogenomes are in a relatively wide range from 16 392 to 18 544 bp (Table 1), but on average (17 092 bp) slightly smaller than the two previously sequenced viviparid mitogenomes (*C*. *cathayensis*– 17 157 bp, *B*. *quadrata*– 17 485 bp). However, they are larger than average caenogastropod (11.1–16.7 Kbp) [17, 25, 26] and gastropod (13.7–14.8 Kbp) [24] mitogenomes. The size range of the remaining 36 caenogastropod mitogenomes retrieved from the GenBank (the two Viviparidae not included) was in a relatively narrow range between 15 078 and 16 907 bp, which further confirms that Viviparidae have the largest mitogenomes among the Caenogastropoda. Judging by the two mitogenomes published for this species so far, *B*. *quadrata* possesses the largest caenogastropod mitogenome sequenced thus far and exhibits a notable intraspecific variability in size, attributable to the variability in size of non-coding regions. As in other Caenogastropoda [23, 25, 28, 47, 68], all eight mitogenomes possessed the standard [20] 37 genes: 13 PCGs (*Cytb*, *NAD1*-*6*, *ND4L*, *COX1-3*, *ATP6* and *8*), 22 tRNAs, and two rRNAs (12S and 16S) (Fig 1; S1 File). Gene strand distribution is almost identical to other Caenogastropoda [17, 26]: all genes are encoded on the major strand, with the exception of the seven tRNAs making up the MYCWQGE box (or its variation WQGEMYC). Whereas tRNA-T can be found on the minor strand in some caenogastropod species [17, 26], it was found only on the major strand in the studied Viviparidae. The length of the 13 PCGs was highly conserved among the eight species: ten genes were distributed within a 12 bp range, with all species sharing an identically sized *NAD2* of 1056 bp. Only *ATP6* exhibited a large variation in size, ranging from 571 bp in *V*. *chui* to 720 bp in *M*. *monodi* (S1 File).

Nucleotide composition of the eight newly sequenced mitogenomes was strongly biased towards A+T (72.5% on average). It was the lowest in *V*. *chui* (69.4%) and the highest in the two *Bellamya* species (74.8–9%) (Table 1). It was somewhat higher for tRNA genes (73.3%) than for the rest of the genome. This (A+T bias) is higher than in other Caenogastropoda [25, 26, 69], as well as the entire Gastropoda class, where the A+T content was reported to vary from 55 to 67% [24]. The A+T values of the remaining 36 (non-viviparid) caenogastropod sequences retrieved from the GenBank for this study (S2 File) ranged from 59.4 (*Ceraesignum maximum*) to 72.7% (*Naticarius hebraeus*) (avg. 67.1%), which further confirms that Viviparidae exhibit an unusually high A+T bias. Intriguingly, *C*. *ussuriensis* exhibited a very high A+T value (74.6%), much closer to the two *Bellamya* species (74.85% avg.) than to other *Cipangopaludina* species (71.5% avg.), which was also reflected in the phylogenetic analysis, where it clustered within the *Bellamya* clade (Fig 1).

Overlapping adjacent genes are common in gastropod mitogenomes, but the overlaps almost always involve tRNA genes [24–26]. These newly sequenced viviparid mitogenomes fit this pattern, as overlaps were found in all eight of them, and most of them involved a tRNA gene (S1 File). Overlap of 1 bp found between *NAD2* and *COX1* in all sequences, apart from *C*. *ussuriensis*, can be attributed to ambiguous annotation, as there is no overlap if *NAD2* uses an incomplete TAA stop codon [20, 70]. The remaining two overlaps between PCGs, *ATP8*/*ATP6* and *NAD4L*/*NAD4*, are both common in Caenogastropoda [17, 26]. The *ATP8*/*ATP6* overlap (4 bp) was not found only in the two *Bellamya* species and *V*. *chui*, whereas *NAD4L*/*NAD4* was found only in these three species (16 bp in *Bellamya* and 7 bp in *V*. *chui*). The tRNA-Ser/*Cytb* overlap (1–3 bp) was also observed only in these three species. The two overlaps between *12S*/tRNA-Val/*16S* were observed in all sequences, however. The first overlap (*12S*/tRNA-Val) was very similar in all eight species (5 to 6 bp), but the second overlap (tRNA-Val/*16S*) was very variable: from 3 to 25 bp. In *C*. *ussuriensis*, this overlap (4 bp between tRNA Val and *16S*) was much more similar to the two *Bellamya* species (3 bp) than to the remaining *Cipangopaludina* species (24 bp). *Cipangopaludina dianchiensis*, on the other hand, exhibited the overlap (25 bp) almost identical to the two *Margarya* species and *C*. *cathayensis* [28], which was also reflected in phylogenetic analysis, where it clustered within the *Margarya* clade. The comparison with previously studied mitogenomes [17, 25, 26] reveals exceptional variability in gene overlaps among gastropod mitogenomes. It should be noted, however, that this is highly likely to be a consequence of annotation artefacts (particularly the overlaps between tRNA Val and *16S*), as the annotation of these eight genomes varied depending on the template used.

Typically for gastropods [17, 25, 26], nearly all genes of the newly sequenced mitogenomes used ATG as the start codon. Exceptions were *ATP6*, which utilised ATA in two *Cipangopaludina* (ATG in *C*. *ussuriensis*) and *Margarya* species, *Cytb*, which used ATA in *B*. *aeruginosa* and ATT in *V*. *chui*, and *NAD4*, which often uses non-standard start codons in Gastropoda [17, 25, 71]. This was evident in the eight sequenced viviparids, none of which used the standard ATG start codon; GTG, ATA, TTG, ATG and ATC were observed instead (S1 File). Terminal codons were also typical for Gastropoda [17, 24–26, 28]: all genes in all eight species terminated with either TAG or TAA stop codon, the latter including the truncated versions of it observed in *ATP6*, presumed to be polyadenylated to TAA [20, 70, 72]. Intriguingly, whereas Grande *et al*. [24] found that *COX3* gene ends with the incomplete stop codon T—in all five gastropod mitogenomes sequenced for their study, we have found it only in *ATP6* of *V*. *chui* and *C*. *dianchiensis* mitogenomes.

### Gene order

**Fig 2:**
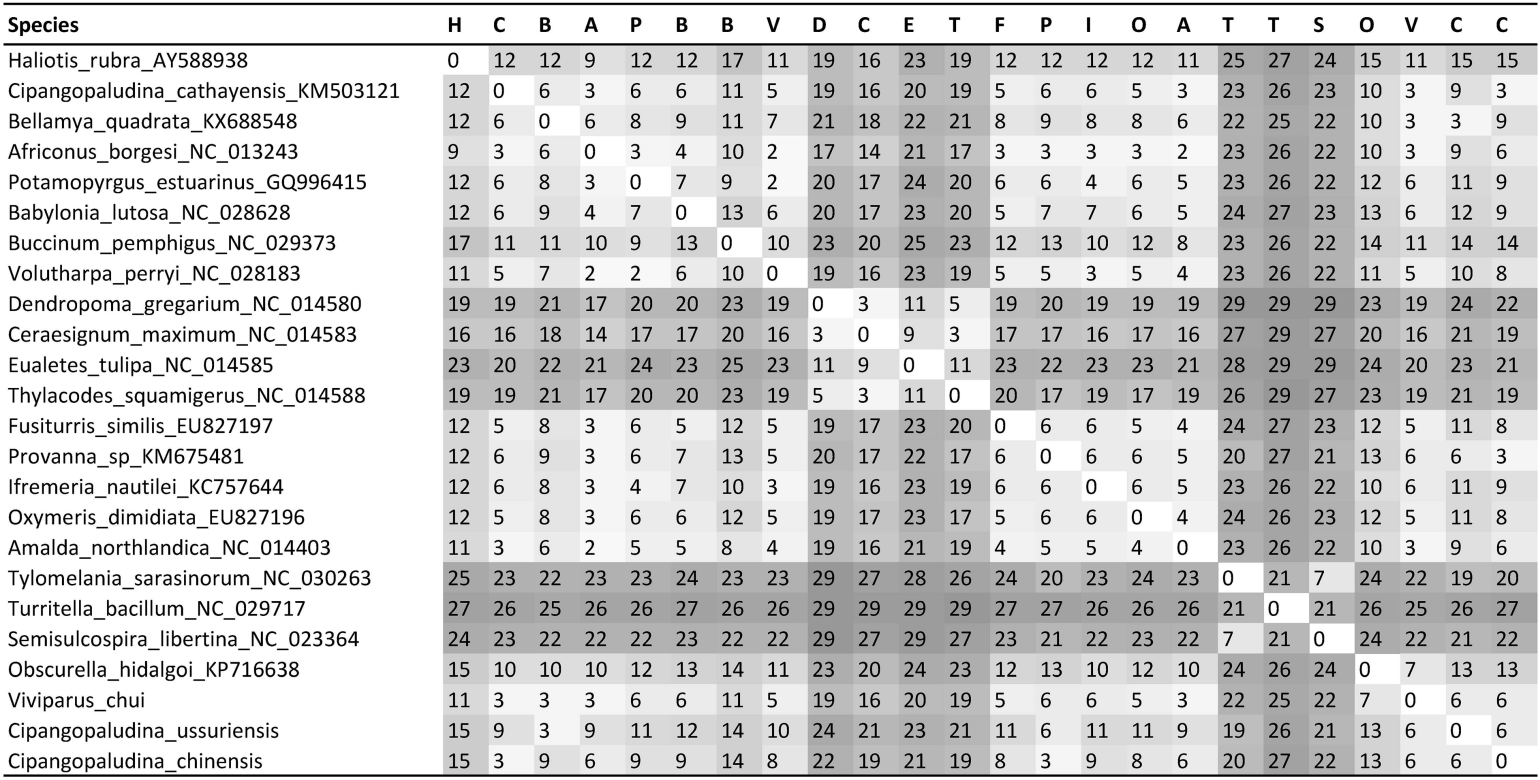
Distance matrix of dissimilarity values inferred using gene orders of the analysed caenogastropod mitogenomes. Where several species shared an identical gene order (see S3 File, Fig 1, and discussion for details), only one species was randomly chosen to represent it. The similarity between the gene orders of a pair of sequences is indicated both by the number (the lower the number, the higher the similarity) and the hue of the matrix field (the lighter the hue, the higher the similarity). Each species from the row heading is represented by its first letter in the column heading.

Gastropod mitochondrial genomes exhibit unusually great variety of gene orders in comparison to mitochondrial genomes of vertebrates [24]. It has been suggested that the mitogenome of the abalone *Haliotis rubra* [61] represents the ancestral gene order of gastropods, albeit with two derived changes [24]. In comparison to this (almost) ancestral gene order, the majority of caenogastropod mitogenomes are believed to share an inversion of a block of 23 genes spanning from *tRNA-F* to *tRNA-E* in *Haliotis*, with a reversion (to the original strand) of the MYCWQGE block [26]. Our analysis confirms that the majority of sequenced caenogastropod mitogenomes possess this highly conserved gene order, with minor variations only in the positions of some tRNAs (Fig 1). Among these, all eleven Viviparidae sequences (nine species) included in the analysis shared an almost identical gene order. The exceptions were a rearrangement of the standard caenogastropod MYCWQGE box (between *ATP6* and *12S*) into a WQGEMYC box found in *Bellamya* + *C*. *ussuriensis* clade (named *Bellamya* clade onwards), and the order of two tRNAs between *NAD5* and *COX3* genes: -T-F- in *Margarya* + *Cipangopaludina* clade, and -F-T- in the *Bellamya* clade and *V*. *chui*. Both rearrangements required only one transposal (MYC-WQGE → WQGE-MYC). The majority of the remaining species (excluding Vermetidae and Cerithioidea) also exhibited a remarkably conserved gene order, as minor discrepancies were observed in only four species (among the 29 species, classified into 22 families and 12 superfamilies): *Ifremeria nautilei* exhibited a rearrangement of the MYCWQGE box into MWYCQGE (requiring only a transposition of W), *Fusiturris similis* a transposition of S2 upstream of *Cytb*, *Oxymeris dimidata* a transposition of V between S2 and T, and *Obscurella hidalgoi* exhibited a number of rearrangements, including the transposition of D upstream of *ATP8*, a reverse transposition of T from the minor strand (between S2 and *NAD4L*) to the major strand between F and *COX3* and a rearrangement of the MYCWQGE box into MQGCWYE (which either required four reversals or two transposals). Interestingly, this gene arrangement was very similar to the one observed in some Viviparidae (Fig 1), which was also reflected in identical gene order similarity values (Fig 2).

This study confirmed that Vermetidae family and Cerithioidea superfamily possess gene orders very different form the remaining Caenogastropoda [26, 57]. Whereas Vermetidae retain relatively high similarity to *H*. *rubra* (Figs 1 and 2), the farthest derived gene order, marked by a number of rearrangements in comparison to *H*. *rubra*, was observed in the Cerithioidea superfamily, comprised of Semisulcospiridae, Pachycilidae and Turritelidae families in this analysis (Figs 1 and 2). However, the gene order among the three Cerithioidea species included in the analysis was highly conserved, with only *Semisulcospira libertina* exhibiting a rearrangement that required a transposition of tRNA-R, reverse transposition of tRNA-Q to the minus strand between -R and -K, and reversal of L2 to -L2 (the latter might be just an annotation artefact, though). Similarly, *Turitella bacillum* exhibited an unusual distribution of genes among strands (Fig 1), but as the order was identical to *Tylomelania sarasinorum*, we suspect that this might be an annotation artefact as well. To further corroborate this, as the sequence remains unpublished, we have re-annotated the entire *T*. *bacillum* CANWEYRQK tRNA box in ARWEN. The analysis confirmed that it was an annotation mistake, and thus the entire box (and we strongly suspect the remaining ‘problematic’ tRNAs) should be re-annotated to the complementary strand. Within the Vermetidae clade, gene order was somewhat less conserved, with all species exhibiting varying numbers of tRNA rearrangements, but only *Dendropoma gregarium* exhibited a different PCG order, with the transposition of *NAD6* upstream of *16S*.

Gene order distance matrix (Fig 2) indicates that the order shared by the majority of species (18, represented by *A*. *borgesi* in the figure) included in the analysis is the closest (dissimilarity index = 9) to *H*. *rubra* (the ancestral state; Fig 1, S3 File). Among the Viviparidae, *V*. *chui* was the closest to *H*. *rubra*, with dissimilarity index of 11, whereas the remaining seven newly sequenced mitogenomes all exhibited the same value (15). As expected, the two clades that exhibited a gene order very different from the remaining Caenogastropoda also had much higher dissimilarity index values: Vermetidae from 16 to 23, and Cerithioidea from 24 to 27. Intriguingly, this indicates that gene order produces a different phylogenetic signal from the nucleotide sequence-based analysis, which produced a topology (Fig 1) that shows Vermetidae as the most basal Caenogastropod group, and Cerithioidea basal to Hypsogastropoda, the group that contains the majority of the diversity of caenogastropods [1, 17]. These results indicate that gene order has a limited usability for inferring the phylogeny of Caenogastropoda. This was observed before, and is believed to be a consequence of the existence of so-called ‘gene order rearrangement hotspots’, around which convergent gene rearrangements are likely [73–78].

### Phylogenetic analysis

Two major groups are recognized within Caenogastropoda: Architaenioglossa (comprising Ampullarioidea, Viviparoidea and Cyclophoroidea) and Sorbeoconcha (comprising Cerithioidea, Campaniloidea and Hypsogastropoda) [1, 17]. Traditionally, based on morphological characteristics, Chinese Viviparidae (Architaenioglossa) species were taxonomically classified into nine genera: *Viviparus*, *Filopaludina*, *Cipangopaludina*, *Margarya*, *Angulyagra*, *Mekongia*, *Rivularia*, *Siamopaludina* and *Bellamya* [4]. Molecular phylogenetic research in the past decade indicated that *Margarya* can be subdivided into two or three separately evolved groups of species, and that some of the *Margarya* species were very closely related to some *Cipangopaludina* species [8, 13, 14]. However, as the molecular markers used in these studies were usually just segments of genes, the resolution provided by these markers may have been insufficient to infer the relationships among these species with high precision.

Both approaches used in our study produced almost identical tree topologies, and thus only the ML tree is shown (Fig 1). Statistical support for the topology was generally rather high, with BI producing higher support values. Minor discrepancies in topology were observed only within the *Bellamya* clade, where the BI analysis showed *B*. *aeruginosa* and *B*. *quadrata* KX688548 to be sister clades (0.88 pp), and in the Cerithioidea clade, where BI analysis showed *T*. *bacillum* and *S*. *libertina* as sister clades (0.97 pp). In both analyses, Viviparidae were divided into three main clades: *Margarya* and *Cipangopaludina* in the first, *Bellamya* and *C*. *ussuriensis* in the second, and *V*. *chui* as the sole representative of the third clade (Fig 1). Paraphyly of *Margarya* has been observed before, using two mitochondrial gene fragments (*COXI* and *16S* rRNA) to infer the phylogeny [8, 14]. All these studies (including our study) are congruent in proposing very close relationships between *C*. *chinensis*, *C*. *dianchiensis*, *M*. *monodi* and *M*. *melanioides*. Although *C*. *cathayensis* sequences were not used in the remaining two studies [8, 14], a number of *Cipangopaludina* sp. sequences were also placed in the same clade. This is rather surprising as *Margarya* is morphologically very distinct from other S-E Asian viviparid genera, but multiple origins of *Margarya* species were proposed a long time ago based on fossil data [79]. This is a strong indication of widespread phenotypic homoplasy in this family. Morphology has been discussed at some length by Du *et al*. [8]. Thus, in congruence with previous studies, our results present further evidence that two *Cipangopaludina* (Hannibal 1912) species included in this analysis, *C*. *cathayensis* and *C*. *dianchiensis* (and possibly also other *Cipangopaludina* species, not included in this analysis [30]), should be renamed into the senior genus *Margarya* (Nevill 1877). However, it should be noted that all these studies [8, 14] have used mtDNA to infer phylogenetic relationships, and thus the hypothetical possibility of a mitochondrial introgression event [80] in the evolutionary history of some of these species should be excluded before officially proposing taxonomic changes. Hence, another study, based on nuclear phylogenetic markers, will have to corroborate these results.

A species not included in Du’s analysis, *C*. *ussuriensis*, exhibited a highly supported sister-clade relationship with the three included *Bellamya* (Jousseaume 1886) sequences. A much larger number of potentially closely related Viviparidae species would have to be included into the analysis to ensure the phylogenetic resolution high enough to precisely determine the relationships between these two clades. However, our results indicate that, if the senior species *C*. *chinensis* (Gray 1834) proves to be a valid representative of the genus, *C*. *ussuriensis* (Gerstfeld 1859) may have to be reassigned to a different genus: possibly Bellamya, possibly a genus not represented in this analysis, or even a completely new genus.

Regarding the remaining sequences, the analysis supports the monophyly of the remaining included families. Among the superfamilies, though, paraphyly is still rather pervasive: Muricoidea superfamily was paraphyletic, with Olivoidea (Olividae) superfamily nested in the middle, which was also observed by Osca *et al*. [17]. The topology actually indicates that Olividae and Babylonidae are sister clades, forming a superfamily together, whereas Volutidae family should belong to a different superfamily. It should be noted that the ML analysis produced a relatively low statistical support for this part of the tree. The topology does not support the placement of Cancellariidae into a separate superfamily (Cancellarioidea); instead they form a monophyletic clade with two Tonnoidea families: Ranellidae and Cassidae. This topology was also observed previously by Osca *et al*. [17], but with low statistical support. In our study, bootstrap support value (ML) was low (35), but BI produced a high pp value of 1.0. Our results support the monophyly of Conoidea superfamily, with four families included in this study (Conidae, Clavatulidae, Terebridae and Turridae) forming a clade. The monophyly of Buccininoidea is also supported, with Buccinidae and Nassaridae forming a clade; as is the monophyly of Cerithioidea, with Pachychilidae Turritellidae and Semisulcospiridae forming a clade. Furthermore, the topology indicates that four superfamilies, Truncatelloidea, Naticoidea, Littorinoidea and Abyssochrysoidea, also form a monophyletic clade. Finally, Viviparidae and Ampullariidae formed a monophyletic Ampullaroidea superfamily.

## Conclusions

Sequencing and analysis of the complete mitogenomes of eight viviparid snail species undertaken here show that some Viviparidae possess a unique gene order (within the Caenogastropoda), marked by a rearrangement of the standard caenogastropod MYCWQGE box. Their genomes are also larger and exhibit a higher A+T bias than most other (caeno)gastropod mitogenomes. Our results present further evidence that at least three *Cipangopaludina* species should be renamed into the senior genus *Margarya*. Furthermore, gene order, A+T contents, gene overlap pattern and phylogenetic analysis all support the hypothesis that *C*. *ussuriensis* is closely related to the *Bellamya* genus, but the phylogenetic resolution achieved, limited by a low number of available sequences, was insufficient to assess whether it should be renamed to *Bellamya ussuriensis* or some other genus. Furthermore, as *Margarya* is morphologically very distinct from the phylogenetically closely related *Cipangopaludina* species, this interesting finding indicates that this group of animals may represent a good model to study the genetic basis for phenotypic diversity.

In order to effectively protect the rapidly diminishing Viviparid snail populations in Yunnan province, it is necessary to resolve the taxonomic status of several currently accepted species. As phenotypic homoplasy appears to be widespread among several viviparid genera, morphology is a rather unreliable tool for resolving the phylogenetic relationships within the Viviparidae. Thus, further molecular phylogenetic studies are urgently needed. Ideally, they should comprise the majority of Chinese viviparid species and use both mitochondrial and nuclear markers, in order to exclude the possibility of mitochondrial introgression.

## Supporting information

S1 Fig Sampling locations in China. (PDF)

S1 File Gene tables for eight newly sequenced viviparid mitogenomes. (XLSX)

S2 File Statistics for all mitogenomes used in this study. (XLSX)

S3 File CREx distance matrix log file. (DOCX)

S1 Table Diagnostic morphological features for the eight studied snail species. (DOCX)

S2 Table Primer pairs used for amplification and sequencing of the studied mitogenomes. (DOCX)
