# Supplementary figures and images for: Sequencing of the complete mitochondrial genomes of eight freshwater snail species exposes pervasive paraphyly within the Viviparidae family (Caenogastropoda)
Source: PLoS One. 2017 Jul 25;12(7):e0181699. doi: 10.1371/journal.pone.0181699 (PMC5526530; doi:10.1371/journal.pone.0181699)

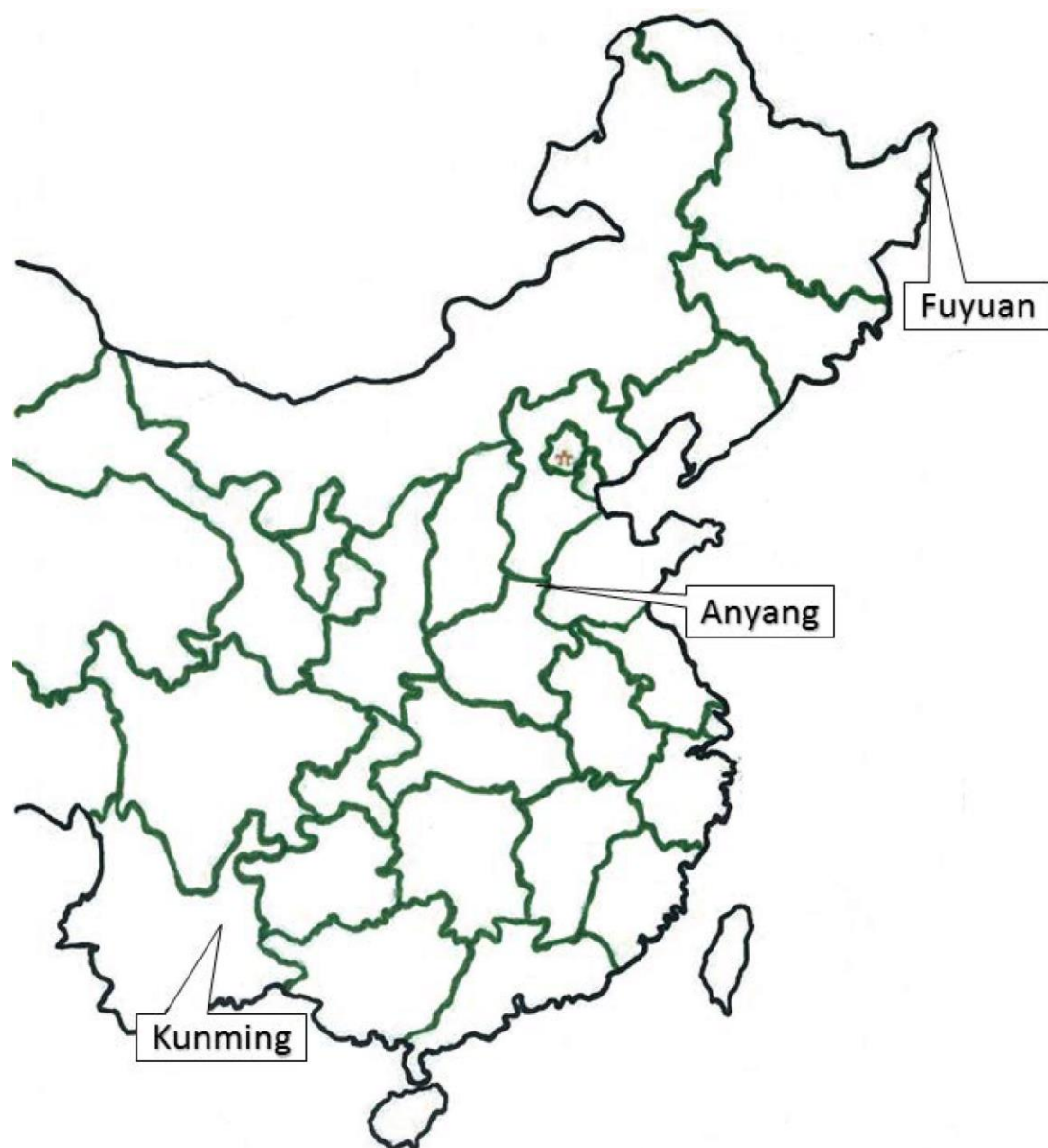

S1 Fig. Sampling locations in China.

Supplement: S1 Fig — (PDF) [file pone.0181699.s001.pdf]
