# Supplementary material for: Sequencing of the complete mitochondrial genomes of eight freshwater snail species exposes pervasive paraphyly within the Viviparidae family (Caenogastropoda)
Source: PLoS One. 2017 Jul 25;12(7):e0181699. doi: 10.1371/journal.pone.0181699 (PMC5526530; doi:10.1371/journal.pone.0181699)
Supplement: S3 File — (DOCX) [file pone.0181699.s004.docx]

**S3 File. CREx distance matrix log file.**

Where several species share an identical gene order, only one species was randomly chosen to represent it.

- Cipangopaludina_chinensis represents Cipangopaludina_dianchiensis, Margarya_monodi and Margarya_melanioides.
- Cipangopaludina_ ussuriensis represents Bellamya_aeruginosa and Bellamya_quadrata.
- Cipangopaludina_cathayensis_KM503121 represents Cipangopaludina_cathayensis_KX688549.
- Potamopyrgus_antipodarum_GQ996416 represents Potamopyrgus_estuarinus_GQ996415.
- Africonus_borgesi_NC_013243 represents Oncomelania_hupensis_FJ997214, Cymbium_olla_EU827199, Cancellaria_cancellata_NC_013241, Naticarius_hebraeus_KP716634, Strombus_gigas_KM245630, Galeodea_echinophora_KP716635, Cymatium_parthenopeum_EU827200, Littorina_saxatilis_KU952094, Lophiotoma_cerithiformis_DQ284754, Varicinassa_variciferus_KM603509, Nassarius_reticulatus_EU827201, Marisa_cornuarietis_NC_025334, Pomacea_maculata_NC_027503, Pomacea_canaliculata_NC_024586, Babylonia_areolata_HQ416443, Neptunea_arthritica_KU246047, and Menathais_tuberosa_KU747972.

Warnings:

- K2 only occurs in Dendropoma_gregarium_NC_014580
- K1 only occurs in Dendropoma_gregarium_NC_014580
- V1 only occurs in Thylacodes_squamigerus_NC_014588
- V2 only occurs in Thylacodes_squamigerus_NC_014588
- K does not occur in Dendropoma_gregarium_NC_014580
- V does not occur in Thylacodes_squamigerus_NC_014588
- L3 only occurs in Thylacodes_squamigerus_NC_014588
