# Supplementary material for: Sequencing of the complete mitochondrial genomes of eight freshwater snail species exposes pervasive paraphyly within the Viviparidae family (Caenogastropoda)
Source: PLoS One. 2017 Jul 25;12(7):e0181699. doi: 10.1371/journal.pone.0181699 (PMC5526530; doi:10.1371/journal.pone.0181699)
Supplement: S1 Table — (DOCX) [file pone.0181699.s005.docx]

**S1 Table. Diagnostic morphological features for the eight studied snail species.**

| ***M. melanioides*** | ***M.* *monodi*** | ***C. dianchiensis*** | ***C. chinensis*** | ***C. ussuriensis*** | ***B. aeruginosa*** | ***B. quadrata*** | ***V. chui*** |
| --- | --- | --- | --- | --- | --- | --- | --- |
| Shell large, up to about 70 mm in width, greenish or yellowish brown,thick, stout, with turreted spire. | Shell large, conical, always with four spiral keels; irregular, and sparse at lower teleconch and body whorls. | Shell large, thick. Body whorl with 7–12 ridges (3–4 ridges clearly). | Shell large (up to 70 mm), oval, Aperture subcircular, inner lip whitish blue and outer lip black in colour, easily brown. | Shell larger, up to 54 mm, brown–gree-nish, with two or three brown band. | Shell thick, medium, less than 35 mm, conical shape. Body whorl with 3 ridges. Aperture pear-circular. | Shell thick, medium, less than 35 mm, elongated, conical shape. Aperture pear-circular. | Shell very thick, medium, less than 30 mm, spherical, surface smooth, with 3 red-brownish band on body whorls. |
