# Supplementary material for: Sequencing of the complete mitochondrial genomes of eight freshwater snail species exposes pervasive paraphyly within the Viviparidae family (Caenogastropoda)
Source: PLoS One. 2017 Jul 25;12(7):e0181699. doi: 10.1371/journal.pone.0181699 (PMC5526530; doi:10.1371/journal.pone.0181699)
Supplement: S2 Table — (DOCX) [file pone.0181699.s006.docx]

**S2 Table. Primer pairs used for ampliﬁcation and sequencing of the studied mitogenomes.**

| **Region** | **F primer sequence (5’→3)** | **R primer sequence (5’→3’)** |
| --- | --- | --- |
| *COX1* | CTACTAATCATAAGGATATTGG | ATCAGCAGGTAAAATATTATC |
| *12S* | TGGTAAATTTAGTGCCAGCAT | TTACCCTTACTATGTTACGACTTATC |
| *16S* | TTATTCGTATTAGGTGATAGCT | ATTAACTTGATCCTTTCGTAC |
| *Cytb* | ATCTATTTGGTGGAATTTTGG | AAGCAAACAAAAAATACCATTC |
| *COX3* | TCATTTGGTTGAATTTAGTCC | AACAAAAAAAGTCGAACCATA |
| *COX1*-*ATP6* (C.ch,C.di,M.me,M.mo) | ATCAATAATCTCGTTTGTTGCTGT | GTTACTGAAGCATTAGTTACCCACA |
| *ATP6*-trnY (C.ch,C.di,M.me,M.mo) | ACAGTTATCCCCATTAAGATGA | GGAAACAAACCCAGTAAAATC |
| trnM-trnW (C.ch,C.di,M.me,M.mo) | GATTTTACTGGGTTTGTTTCCA | AACCAAATCAGACCAAGTAACTGT |
| trnC-trnQ (C.ch,C.di,M.me,M.mo) | ACAATGATTAAAGGTAAGAGTGGTGT | ATTTCCTTTTCTTACAACGCAAC |
| trnQ-trnE (C.ch,C.di,M.me,M.mo) | CTTTCTGCTTTCTGTATAACGTG | CAAGCGGAAATTTTAATCAAC |
| trnE-12S (C.ch,C.di,M.me,M.mo) | ATTCTTTGTACGCTAATACTGTT | AAATATCGTAAACTCAACCCA |
| 16S-*Cytb* (C.ch,C.di,M.me,M.mo) | CGTGAGCCAGGTCAGTTTCTATC | GAACTAAAAGCCAAATCAATATGTCC |
| *Cytb*-*COX3* (C.ch,C.di,M.me,M.mo) | CTTTTGCCTTTTGTTATTGTAGTG | AAAATAAGCCCAAAAAAAAGC |
| *COX3*-*COX1* (C.ch,C.di,M.me,M.mo) | TGGGTGTTTATTTCACATTTCTCC | CAACCAGTTCCTACACCTCCTTC |
| *COX1*-*ATP6* (B.a,B.q) | TATTGTTTGAGAGGCTTTAGT | TTAGTACAATATGACCTGCA |
| *ATP6*-trnE (B.a,B.q) | CAGTTAGTATTAATGTTCGATC | GTAAAATGTTTGGAAGCACAT |
| trnE-trnM (B.a,B.q) | GAATACTAATAAGAGAAAAGAGC | AATTAGGAACTATAGTAGTTGCA |
| trnM-12S (B.a,B.q) | AAATTAAAAACAGCGCTACCT | AAACTACATACTGGTGTTAGT |
| 12S-16S (B.a,B.q) | TTTTCACTAACACCAGTATGT | GCATAATAATCAAGATTAGCCT |
| 16S-*Cytb* (B.a,B.q) | AGCATAATCTTCTTGGGGAGT | TCAACAGTAAATCCACCTCAAA |
| *Cytb*-*COX3* (B.a,B.q) | AGTTACTCCAGTTCATATCCA | TTGAAGTATCTAAAGGATTACG |
| *COX3*-*COX1* (B.a,B.q) | ACATGAGCCCATCATAGATTA | AATCCTGACCATACACCAAAC |
| *COX1*-16S (V.c) | GTGGTGTTGTTTGAAGGTTAC | TCAGTCGTCATATAAATCTACACT |
| 16S-*Cytb* (V.c) | GATAACAGCATAATCTTCTTTG | CCACCTCAGACTCACTCTACTA |
| *Cytb*-*ND5* (V.c) | TTATGCTATTTTACGGTCTATC | ACAATCTTGAATATACCTTGTC |
| *ND5*-*COX3* (V.c) | TTAGTTAAAGTGGCTACAACA | CAACAAGATCCTAATTCTAATC |
| *COX3*-*COX1* (V.c) | GGGCTGGGTGTTTATTTTACT | TTGACCAAACTCAGCACGA |
| *COX1*-16S (C.u) | TAGAGTCATTGATTTCGTTTG | ACCAAGATTAGCCTTCATTAT |
| 16S-*Cytb* (C.u) | ATAGTTACCATAGGGATAACAGC | CCACAAGTATTGAGTGATTCTG |
| *Cytb*-*COX3* (C.u) | AGAAAGAAAAAGACCAGTAAC | GTGCCATCATTGAAATATAG |
| *COX3*-*COX1* (C.u) | CTGGATTATGCTGACCTCCTGTA | AATGCAGAAAAGCCATATTAGGAG |

For primer pairs which were not used for all eight sequenced mitogenomes, abbreviated name of the species for which the primers were used is indicated in the brackets.
